# Use of Spirometry for Diagnosis and Management of Chronic Obstructive Pulmonary Disease: An Approach of General Practitioners and Pulmonologists From Karachi, Pakistan

**DOI:** 10.7759/cureus.97998

**Published:** 2025-11-28

**Authors:** Fatima Zaina, Ayesha Akhtar, Jamil Muqtadir, Ahmed Wahab, Gohar Fatima

**Affiliations:** 1 Pulmonology, Dr. Ziauddin Hospital, Karachi, PAK; 2 Infectious Diseases, Dr. Ziauddin Hospital, Karachi, PAK; 3 Medicine, Dr. Ziauddin Hospital, Karachi, PAK; 4 Pulmonology and Critical Care, Liaquat National Hospital, Karachi, PAK

**Keywords:** asthma, copd, general practitioner, pulmonologists, spirometry

## Abstract

Background: Spirometry is a quick and non-invasive method for evaluating an individual's lung health. Respiratory illnesses, including chronic obstructive pulmonary disease (COPD) and asthma, are screened for, diagnosed, and tracked with its help. This study aims to ascertain the diagnosis and therapy of COPD from the perspective of Pakistani general practitioners and consultant pulmonologists in Karachi.

Methods: A retrospective cross-sectional study design was adopted for this research. This study was conducted over six months, from January 2020 to June 2020. The sample consisted of general practitioners and pulmonologists from all campuses (North, Clifton, and Kemari) of Dr. Ziauddin Hospital, Karachi, Pakistan.

Results: The usage of spirometry, a key tool in COPD diagnosis and management, was found to be low, with only 13.6% of patients having undergone the test before or during treatment. The rate of spirometry use was 37.5% among pulmonologists and a mere 10.3% among non-pulmonologists. This indicates a significant gap in the use of this important diagnostic tool. Oral bronchodilators and long-acting beta-agonist (LABA)/steroids were more commonly prescribed by pulmonologists, with a frequency of 87.5% and 37.5%, respectively, compared to 41.4% and 20.7%, respectively, among non-pulmonologists.

Conclusion: Spirometry, the recommended test for the diagnosis of several respiratory diseases, including COPD, is not used as frequently as it should be. The study found that non-pulmonologists are more reluctant to use spirometry than pulmonologists. This emphasizes the necessity of raising awareness and providing training regarding the significance of spirometry in the identification and treatment of COPD.

## Introduction

Respiratory illnesses are the primary cause of morbidity and mortality globally. Asthma affects 235 million people worldwide, and chronic obstructive pulmonary disease (COPD) causes over 3 million fatalities, according to the WHO. Furthermore, low- to middle-income nations account for more than 90% of those fatalities [1]. The term "Chronic Obstructive Pulmonary Disease" shelters several lung diseases, including emphysema and chronic bronchitis; the downfall of this disease is that lung damage is not fully reversible [2]. Most commonly, the lungs are the prime target of chronic obstructive pulmonary disease (COPD). Still, with extensive research on this topic, it is also established that it is a complex multicomponent disease characterized by chronic systemic inflammation and often accompanied by multiple comorbidities, which can significantly affect the outcome of the disease [3].

A straightforward, noninvasive test to evaluate an individual's lung function is spirometry. It is employed in the screening, diagnosis, and surveillance of respiratory conditions such as asthma and COPD [4]. Age, sex, height, weight, ethnicity, smoking habits, physical fitness, and environmental conditions can all influence spirometry results [5,6]. COPD is diagnosed as chronic airflow obstruction (forced expiratory volume in 1 second (FEV_1_)/forced vital capacity (FVC) post-bronchodilator ratio <70%) [7].

Multiple societies have published international guidelines to manage COPD, including symptoms, diagnostic approaches, and treatment regimes. These guidelines have declared spirometry as a gold-standard test for the diagnosis of COPD. Sadly, many studies have been published in various international journals that state that these guidelines are rarely followed by primary care physicians, even though they are readily available [8]. According to our best knowledge, no known published literature is available regarding the diagnostic approaches of general practitioners and pulmonologists in most of the areas of Karachi, Pakistan, for COPD. Our primary objective is to determine the doctors' strategy for COPD diagnosis and management in Karachi, Pakistan.

## Materials and methods

A retrospective cross-sectional study design was adopted for this research. This study was conducted for six months from January 2020 to June 2020; the sample consisted of general practitioners and pulmonologists from most of the areas of Karachi, Pakistan. The sample size was calculated at a 95% confidence interval with a 5% margin of error and was found to be 230. This research was done in accordance with the Helsinki Declaration.

Inclusion criteria included all the general practitioners with registered MBBS degrees currently treating patients in OPDs and pulmonology consultants working across the North, Clifton, and Kemari campuses of Dr. Ziauddin Hospital, Karachi. Exclusion criteria include all doctors of other specialties and those who refuse to consent. The questionnaire consisted of three sections: the first section had questions relating to demographic information; the second section consisted of questions related to knowledge, awareness, indication, and uses of spirometry; the third section included questions about the treatment they are currently using for COPD. The questionnaire was tested on a sample of 20 people, and Cronbach's alpha was found to be 0.807. The questionnaire was distributed by hand, and later, data were coded for further analysis.

To analyze the data, the Statistical Package for the Social Sciences (SPSS) version 22 (IBM Corp., Armonk, NY) was utilized. With reference values established and mean±2 SD deemed significant by means of the standard distribution curve, the quantitative variables were presented as mean and standard deviation. In parallel, percentages were used to characterize qualitative factors. Chi-square was used to establish correlations with other variables; a p-value of less than 0.05 was deemed statistically significant, along with a 95% confidence range.

## Results

A total of 264 patients receiving treatment for underlying pulmonary disorders were included, out of whom 65.2% (n = 172) were diagnosed with asthma, and 33.3% (n = 88) were diagnosed with COPD. The mean age was 59.14 ± 14.3 years, and the mean treatment duration was 6.05 ± 8.03 years. Only 32 (12.1%) were under treatment with pulmonologists, and the remaining 87.9% (n = 232) of the patients were receiving treatment from non-pulmonologists. Spirometry either before or during treatment was performed only in 13.6% (n = 36) of the patients, and the rate of use of spirometry was significantly higher for pulmonologists as compared to non-pulmonologists, with the frequency of 37.5% (n = 12) vs. 10.3% (n = 24); p < 0.001. Demographic and clinical characteristics stratified by the subspecialty of the treating doctor are presented in Table 1. Chi-square and t-test values are presented in Tables 1, 2, with p <0.05 considered statistically significant.

**Table 1:** Demographic and clinical characteristics stratified by the subspecialty of the treating doctor *p-Value <0.05 is statistically significant. COPD, chronic obstructive pulmonary disease.

Characteristics	Total (n = 264)	Pulmonologist (n = 32)	Non-pulmonologist (n = 232)	Test statistic	p-Value
Gender (male/female)	50%/50%	50%/50%	50%/50%	χ² = 0.00	0.99
Age (years, mean ± SD)	59.14 ± 14.3	58.75 ± 8.45	59.19 ± 14.94	t = 0.16	0.871
Up to 50 years	21.2%	12.5%	22.4%	χ² = 1.65	0.198
More than 50 years	78.8%	87.5%	77.6%	—	—
Diagnosis (asthma/COPD/none)	65.2%/33.3%/1.5%	62.5%/37.5%/0%	65.5%/32.8%/1.7%	χ² = 0.78	0.677
Treatment duration (years, mean ± SD)	6.05 ± 8.03	2.88 ± 0.94	6.5 ± 8.48	t = 2.45	0.017*
Up to 5 years	65.2%	100%	60.3%	χ² = 18.9	<0.001*
5-10 years	22.7%	0%	25.9%	—	—
More than 10 years	12.1%	0%	13.8%	—	—
Spirometry before or during treatment	13.6%	37.5%	10.3%	χ² = 12.6	<0.001*

**Table 2:** Treatment regimens used by pulmonologists and non-pulmonologists *p-Value <0.05 is statistically significant. SABA, short-acting beta-agonists; LABA, long-acting beta-agonists; LAMA, long-acting muscarinic antagonists.

Treatment regimen	Total (n = 264)	Pulmonologist (n = 32)	Non-pulmonologist (n = 232)	Test statistic	p-Value
SABA	45.5%	37.5%	46.6%	χ² = 0.93	0.335
SABA/Steroids	6.1%	12.5%	5.2%	χ² = 2.64	0.103
LABA/Steroids	47.0%	87.5%	41.4%	χ² = 12.15	<0.001*
Nebulization	54.5%	62.5%	53.4%	χ² = 0.93	0.335
LAMA	19.7%	12.5%	20.7%	χ² = 1.19	0.275
Oral bronchodilators	22.7%	37.5%	20.7%	χ² = 4.56	0.033*
Inhaled steroids	4.5%	0.0%	5.2%	χ² = 1.74	0.188

Treatment regimens stratified by the subspecialty of the treating doctor are presented in Table 2. Nebulization was prescribed to a majority of the patients, 54.5% (n = 144), long-acting beta-agonists (LABA)/steroids were prescribed to 47% (n = 124), short-acting beta-agonists (SABA) to 45.5% (n = 120), and oral bronchodilators were prescribed to 22.7% (n = 60) of the patients. Prescription of oral bronchodilators and LABA/steroids was significantly more prevalent among pulmonologists as compared to non-pulmonologists with a frequency of 87.5% (n = 28) vs. 41.4% (n = 96), p < 0.001 and 37.5% (n = 12) vs. 20.7% (n = 48), p=0.033 for oral bronchodilators and LABA/steroids respectively, while prescription of SABA and long-acting muscarinic antagonists (LAMA) was relatively more prevalent among non-pulmonologists.

## Discussion

When assessing a number of respiratory conditions, including asthma, emphysema, and chronic bronchitis, spirometry is a crucial diagnostic tool [9,10]. The GOLD guidelines recommend the use of spirometry for patients who are 40 years and above and have a history of smoking, along with a family history of respiratory diseases. Although several guidelines and studies have identified spirometry as the gold standard for diagnosing and following several respiratory diseases, many doctors still do not use it. According to our research, spirometry was performed before or during treatment in only 13.6% of the patients. The use rate of spirometry was significantly higher for pulmonologists compared to non-pulmonologists, with a frequency of 37.5% vs. 10.3%. In Italy, general practitioners are particularly reluctant to carry out spirometry for the diagnosis of COPD, even though they are well aware of its increasing burden [11]. Another study carried out in Australia showed that 47% were diagnosed with COPD by general practitioners, but out of these, only seven were diagnosed through spirometry. General practitioners also don’t support the use of spirometry for management. Fifty-eight percent of patients had spirometry for management, but only 32% had it done by general practitioners [12]. In the USA, it was reported that for the diagnosis of asthma, only 23% of spirometry was carried out by general practitioners compared to 80% carried out by pulmonologists [13]. The use of spirometry in primary health care was limited for the screening of smokers who haven’t developed symptoms [14]. This practice was supported by several authors who believed that spirometry should not be used for smokers without symptoms and that smoking should be discouraged in all smokers [15,16].

There are several reasons for general practitioners' underuse of spirometry. One of the main reasons is the need for more knowledge about the role of spirometry in diagnosing respiratory diseases, especially among junior doctors. Thus, seniors should emphasize and educate the junior doctors [17]. Other reasons for underuse include limited availability of spirometers (25%), lack of knowledge about spirometers in the diagnosis of respiratory diseases (65%), and inability to understand spirometry results (81%) [18]. These barriers have greatly limited the use of spirometry. Therefore, training programs and workshops should be carried out to bring change in these practices. The indications of spirometry, as recommended by the international guidelines, should be taught to change doctors' perceptions, particularly general practitioners. Similarly, the interpretation of spirometry results should be part of the curriculum so that future doctors are confident enough to understand and interpret the results.

Encouraging the use of spirometry will enable the early detection of respiratory diseases. In the case of COPD, this will allow for the minimization of risk factors like smoking, which can slow down the progression of airway obstruction. More importantly, it will enable the optimization of treatment, leading to a reduction in symptoms and an improvement in the quality of life for patients [19].

This study also revealed that prescription oral bronchodilators and LABA/steroids were significantly more prevalent among pulmonologists than non-pulmonologists, with a frequency of 87.5% (28) vs. 41.4% (96). Meanwhile, prescriptions of SABA and LAMA were relatively more prevalent among non-pulmonologists. Another study carried out in the Netherlands showed that general practitioners used spirometry to follow up after treatment with steroids [14]. The limitations of this study include a small sample size.

## Conclusions

Spirometry is the recommended test for the diagnosis of several respiratory diseases. Prompt diagnosis allows early disease management by minimizing risk factors and appropriate medications. Unfortunately, spirometry is underused, especially among non-pulmonologists.
